# Institutional and Social Issues Surrounding Genetic Counselors in Japan: Current Challenges and Implications for the Global Community

**DOI:** 10.3389/fgene.2021.646177

**Published:** 2021-04-13

**Authors:** Yayoi Aizawa, Atsushi Watanabe, Kazuto Kato

**Affiliations:** ^1^Department of Biomedical Ethics and Public Policy, Graduate School of Medicine, Osaka University, Suita, Japan; ^2^Division of Clinical Genetics, Kanazawa University Hospital, Kanazawa, Japan; ^3^Support Center for Genetic Medicine, Kanazawa University Hospital, Kanazawa, Japan

**Keywords:** genetic counselor, genetic counseling, genomic medicine, national qualification, professional development, research, workforce

## Abstract

In recent years, genetic counseling has started playing a major role in the field of genomic medicine. There are currently about 7,000 genetic counselors in more than 28 countries, with 267 certified genetic counselors in Japan alone (about 2 per million population, as of April 2020). While the rapid advancement of genomic medicine has expanded this field, the challenges genetic counselors face are also evolving. This article aims to provide an overview of the institutional and social issues surrounding genetic counselors in Japan and discuss implications for the global community. In Japan, with the rapid changes in genomic medicine and the establishment of a delivery mechanism within the healthcare system, several issues need to be discussed. First, many genetic testing, counseling, and preventive healthcare programs are not covered by public health insurance. Second, reducing human resource shortages for genetic counseling is an urgent issue. Third, the lack of a national qualification in the profession is critically important issue in the field. Fourth, research on the role and value of genetic counselors is still limited. To address these issues, discussions among relevant stakeholders, including genetic counselors, professionals in genomic medicine, and lawmakers, are necessary. Additionally, we believe that research by genetic counselors to evaluate and improve their practice and examine institutional and social issues is crucial for developing their profession’s activities and delivering high-quality healthcare to many people. To establish the position and role of the relatively new profession of genetic counselor, sharing information and collaborating on institutional and social challenges faced by genetic counselors globally will be beneficial.

## Introduction

In recent years, genomics as a specialization in medicine has progressively expanded and attracted attention. Genetic counselors play a major role in genomic medicine. The first genetic counseling training program was established at the Sarah Lawrence College in New York, United States, in 1969. There are about 7,000 genetic counselors in more than 28 countries, as of 2018 (Abacan et al., 2019).

In Japan, there is a certification program called “Certified Genetic Counselor” (CGC), which is a joint qualification obtained under the Japan Society of Human Genetics and the Japanese Society of Genetic Counseling (Japanese Board of Genetic Counseling, 2010). To become a CGC, an individual must graduate from a professional training program approved by the Japanese Board of Genetic Counseling established by the two societies and pass the certification examination. Requirements for the professional training programs include the following: a master’s degree must be awarded; clinical geneticists and/or CGCs should be included in the faculty in charge of the program; and the curriculum must meet the achievement goals set by the Japanese Board of Genetic Counseling. For 7 years from 1998 onward, a research group funded by the national government began studying the training of professionals involved in genetic medicine, including genetic counselors; two training programs were established in 2003 and one in 2004. The first certification examination was conducted in 2005 by the Japanese Board of Genetic Counseling. In designing these systems, that of the United States was used as a reference. Its name was trademarked in 2019. In the almost 15 years since the CGC certification system was established in 2005, it has faced many challenges—such as the lack of acknowledgment as a national qualification and workforce issues related to employment conditions suitable for a master’s degree—as a relatively new profession in the medical field. Even as genomic medicine has rapidly expanded, the challenges they face are also evolving.

Globally, genetic counselors face various institutional and social challenges in their work, and the situation varies across countries (Balobaid et al., 2016; Abacan et al., 2019; Sun et al., 2019; Pestoff et al., 2020). Although there are many commonalities in practice, training curricula, certification, and practice models vary among countries and depend on the country’s healthcare system, laws, and regulations (Abacan et al., 2019). We believe that an overview of the issues in Japan will be not only useful in planning future strategies for improving the quality of the genetic counseling delivery system but also provide valuable information for those who are assessing possible approaches to address issues regarding genetic counseling in other countries. It is also important to share information on the situation in various countries to stimulate global discussions on common issues, such as the role that the profession needs to play in future medicine. This article aims to provide an overview of the institutional and social issues surrounding genetic counselors in Japan and discuss its implications for the global community, especially to utilize the expertise that genetic counselors have in suitable situations.

## Overview of Japan’s Healthcare System

Japan is an island country covering 377,974 square kilometers. It is made up of 47 prefectures and has a population of about 126 million (Government of Japan, 2020), with most of the population concentrated in metropolitan areas such as the capital city, Tokyo. The Japanese medical service sector is based on a universal health insurance system (Ministry of Health, Labour and Welfare, 2012). Patients pay only a portion of their bills for medical treatment (in most cases, the out-of-pocket rate is 30%), with the remaining charges being covered by insurance. However, as a rule, individuals must bear the entire cost of medical treatment when a part of it is not covered by insurance, because the combination of public insurance and privately paid treatment is not allowed except for in a few instances. Medical reimbursements are reviewed and revised every 2 years by the government.

Most medical professions are regulated by qualification laws that define the permitted scope of work. To obtain a license, individuals must complete a required curriculum at an educational institution established in accordance with the law, and pass a national examination. In Japan, these licenses for healthcare workers are granted by Ministry of Health, Labour, and Welfare. In this article, when referring to these systems, we use the term “national qualification.”

There are several government organizations involved in healthcare in Japan; the Ministry of Health, Labour, and Welfare is mainly responsible for administration related to medical care and welfare (Ministry of Health, Labour and Welfare, 2014). In addition, the Ministry of Education, Culture, Sports, Science and Technology is responsible for the education of medical professionals and medical research (Ministry of Education, Culture, Sports, Science and Technology, 2009).

## Rapid Changes in and Future Perspectives on Genomic Medicine

In Japan, as in other countries, there have been rapid advancements in genomic medicine, in line with advancements in genome-wide analysis technology. These changes have been particularly significant during the past few years, with the implementation of various large-scale clinical studies and the launch of new healthcare services related to genomic medicine.

In 2013, American actress Angelina Jolie announced that she had hereditary breast and ovarian cancer syndrome (HBOC) (Jolie, 2013). This caught the attention of many people across the globe, including Japan. The publicity also led to an increased interest in medical care for hereditary cancer, particularly HBOC, among medical professionals. It offered a great opportunity to discuss making medical services for hereditary cancer available to more hospitals. Although most such medical services were out-of-pocket at this point, it led to greater opportunities to consider testing as an option. That year, a clinical study of non-invasive prenatal genetic testing was also initiated in Japan (Samura et al., 2017), and an active debate unfolded on the ideals and pros and cons of such testing including varying opinions regarding the possibility of choosing abortion and importance of providing information about prenatal testing. This discussion continues among various stakeholders, including healthcare professionals, as well as their and patients’ organizations. Both topics received extensive coverage and became major reasons for the recognition of genetic testing and counseling in society. Mainly university hospitals had already established genetic clinics, and the abovementioned events also led to an increase in the number of general hospitals with genetic clinics. With the aim of ensuring cooperation among the various clinical practice sections of medical genetics, the National Liaison Council for Clinical Sections of Medical Genetics was established in 2003. As of 2021, a total of 132 medical institutions—including university hospitals and other medical institutions—had joined (Fukushima and Takada, 2020; National Liaison Council for Clinical Sections of Medical Genetics, 2020).

There have been some major changes in healthcare in Japan during the last few years. In 2018, one of the national healthcare and research projects—known as “cancer genomic medicine,” which utilizes cancer gene panel tests—began to develop a healthcare delivery system for tumor profiling testing, which was covered by public health insurance (Ebi and Bando, 2019). This, in turn, prompted the need to establish a system to address pathological variants in genes causing hereditary cancer on the cancer gene panel, mainly called secondary findings. This should include confirmatory germline testing and genetic counseling. Efforts are being made to improve the system providing medical care for rare diseases, including research projects of exome analysis for undiagnosed diseases funded by the government. The government has also been discussing the introduction of whole-genome analysis for cancer as well as rare diseases mainly in a research context since 2019 (Ministry of Health, Labour and Welfare, 2019).

## Current Challenges in the Implementation of Genomic Medicine in Japan

### Challenges Related to the Delivery System for Genomic Medicine

Genomic medicine requires the provision of individualized prevention and treatment based on appropriate diagnoses for individuals at risk, not merely those with symptoms. Improving healthcare delivery and securing human resources are an important factor in ensuring that such services are available to those who need it.

In Japan, many genetic testing procedures, preventive treatments, and surveillance mechanisms for unaffected individuals are not covered by public health insurance (Aizawa et al., 2020). With the advancement of genomic medicine, the scope of medical care covered by public insurance is gradually expanding, with various discussions and lobbying endeavors by stakeholders. The *BRCA1/2* genetic test was already covered as a companion diagnosis for the PARP inhibitor olaparib in 2018. As a result of the 2020 reforms in the medical reimbursement system, the government committee for national health insurance decided to extend the application of genetic testing to individuals with a family history of HBOC and breast and ovarian cancer patients. Further, reduced-risk mastectomy or risk-reducing salpingo-oophorectomy for cancer patients with *BRCA1/2* associated HBOC are also covered by insurance following these reforms. In the 2020 reforms in the medical reimbursement system, the number of diseases diagnosed by genetic testing covered by public insurance increased by 65—that is, from 75 to 140 intractable diseases—including genetic diseases occurring in children and intractable diseases that are covered by the national subsidy system for healthcare expenses (Table 1). Many genetic tests that are not covered by insurance, such as Lynch syndrome and Familial hypercholesterolemia, are conducted at patients’ own expense or in the course of research. Under the national public insurance system, genetic counseling is only covered by insurance if the patient has undergone a genetic test that insurance permits, and only a limited number of medical facilities are registered to provide this genetic counseling. Prenatal genetic testing and preimplantation diagnoses are not covered by national health insurance. The availability of these services is limited. Preimplantation diagnoses have been applied to disorders with a high risk of seriously affecting children. The Japan Society of Obstetrics and Gynecology and related stakeholders are currently engaged in discussing the review system and the scope of preimplantation diagnosis.

**TABLE 1:** Reimbursement for genetic testing and genetic counseling in Japan.

Revised year	Diseases covered	Medical service fees of genetic testing (Yen)	Additional fees of genetic counseling (Yen)
2008	13	20,000	5,000
2010	15	40,000	5,000
2012	36	40,000	5,000
2014	36	38,800	5,000
2016	72	38,800	5,000
2018	75	38,800/50,000/80,000*^1^	10,000
2020	140*^2^	38,800/50,000/80,000*^1^	10,000

^*1^Categories are defined by interactive disease. ^*2^BRCA1/2 genetic testing (202,000 Yen) for the diagnosis of hereditary breast cancer ovarian cancer syndrome is not included here because it is in a separate category.

Therefore, some of the most crucial information that should be provided to clients during genetic counseling concerns the medical costs, including the genetic testing and the preventive care. The burden of high medical costs outside of national insurance for patients can be a factor in their hesitancy to pursue genetic testing and preventive care, which can be frustrating for medical professionals.

The scope of genetic counseling, including pre-symptomatic patients, continues to expand with the increasing number of medically actionable genetic disorders and the possibility of tumor profiling tests, which can reveal that a patient has an inherited form of cancer. The extent to which genomic medicine should be included in insurance coverage requires further discussion, with reference to the situation in other countries that are actively promoting genomic medicine.

### Challenges Related to the Growing Need for Genetic Counseling Professionals

There is also an urgent need for training of professionals for genetic counseling. Although there are no official data available, the number of genetic counseling cases has steadily increased nationwide during the last decade. This has led to an increase in the number of medical professionals who provide genetic counseling. Educating and supporting other healthcare professionals has also become an important activity for genetic counseling professionals, as genomic information is increasingly handled in clinical practice. Genetic counseling professionals have played a central role in the advancement of genomic medicine, including efforts to establish academic societies and organizations related to genomic medicine (Fukushima and Takada, 2020). Through the efforts of relevant stakeholders, mainly academic societies, basic knowledge related to genetics has been introduced into the curricula of medical school.

In Japan, 1,410 clinical geneticists who are physicians (about 11 per million population, as of July 2020) (Japanese Board of Medical Genetics and Genomics Clinical Genetics, 2015) and 267 CGC who are not physicians (about 2 per million population, as of April 2020) (Japanese Board of Genetic Counseling, 2010) have been listed as genetic counseling professionals in the “Guidelines for Genetic Tests and Diagnoses in Medical Practice” by the Japanese Association of Medical Sciences (2011). The same guidelines state that genetic testing for diagnostic purposes in affected patients should, in principle, be handled by the primary physician, and that genetic counseling is necessary for pre-symptomatic patients or those with a prenatal diagnosis (Japanese Association of Medical Sciences, 2011). In addition, the existence of genetic counseling professionals is essential for the abovementioned “cancer genome medicine.” Requirements for medical facilities to provide insured tumor profile testing, which is designated by the Ministry of Health, Labour, and Welfare, include having at least one physician and one or more expert with genetic counseling skills in the department that provides genetic counseling services (Director of the Health Bureau, Ministry of Health, Labour and Welfare, 2019).

Training genetic counseling professionals has not been able to keep up with the rapid growth in demand. Owing to the practical difficulties of training numerous people at once, each graduate school can only train a few people per year, which results in a shortage of genetic counselors. Further collaboration between stakeholders is necessary, as there is a need to improve the system to provide high-quality genetic counseling promptly to those who need it.

## The Position of Genetic Counselors in the Healthcare System and the Movement Toward a National Qualification

A critical challenge faced by the genetic counseling industry in Japan is the professional qualification of genetic counselors. While other medical professions specializing in genomic medicine—including physicians and nurses—have a national qualification to pursue, CGC is not yet a national qualification but a certification accredited by academic societies. This means that the scope of genetic counselors in the context of the Japanese healthcare system is unclear, whereas the scope of practice of other nationally qualified professionals is clearly defined by law. When the current CGC certification system was established in Japan, it had considerably little recognition in the medical field and offered few opportunities for employment (Kohzaki, 2014). However, the demand for genetic counselors, especially in clinical practice, has grown rapidly, owing to various changes in genomic medicine and genetic counseling in recent years. There is an overwhelming shortage of personnel to meet the demand. There are also regional differences in placement, with many jobs and employment opportunities available in urban areas, and no genetic counselors at all in some prefectures. Further, while the number of job opportunities for genetic counselors has been increasing, especially in the last few years, applications in rural areas are often lacking. Approximately 40% of genetic counselors live or work in the densely populated prefectures such as Tokyo, Aichi, and Osaka. The number of training programs accredited by the Japanese Board of Genetic Counseling has been increasing gradually, and that of new CGCs is also rising annually. As of April 2020, there were 20 training programs across the country, of which 19 were established as master’s programs and only one as a doctoral program (Japanese Board of Genetic Counseling, 2010). The locations of these programs are also imbalanced. Among the 20 programs, about half are in the three large metropolitan areas, Kanto, Tokai, and Kansai which include prefectures of Tokyo, Aichi, and Osaka, respectively.

Globally, the state of genetic counselor certification, legislation, and licensing systems vary. Countries with national and state regulations are limited, such as South Africa, Malaysia, and parts of the United States; Australia is currently lobbying for the regulation of the profession (Ormond et al., 2018; Abacan et al., 2019). In Japan, there have been discussions on national qualifications since the establishment of the certification system by academic societies; however, this is yet to be accomplished. Although many medical professions became national qualifications because of laws developed after World War II, it is not easy for new professions to become national qualifications. In Japan, it took about 50 years of debate to institute the national qualification of “certified public psychologists,” whose primary job is to provide psychological support. There has been little public discussion on the national qualification of genetic counselors. However, the final report based on the interim report of the Council for the Promotion and Realization of Genome Medicine, which was established within the Office of Healthcare Policy of the Prime Minister of Japan and His Cabinet, stated that the need for national qualifications for genetic counselors should continue to be considered, while considering the progress made in the field of genomic medicine, as a long-term issue (Council for the Promotion and Realization of Genome Medicine, 2019). One of the three proposals in the “Development of Systems and Human Resource for Promoting Genomic Medicine” proposal issued by the Clinical Genome Medicine Subcommittee of the Science Council of Japan (2020) is the national qualification of genetic counselors.

With the abovementioned advancements in genomic medicine, the role of the genetic counselor is expanding, as opportunities to handle genome-wide information and areas of engagement are also expanding. The nature of the counselor’s work is changing from genetic to genomic counseling (Patch and Middleton, 2019). There are also certain difficulties because of the varying roles required in different facilities and the lack of professional independence in the system; this emphasizes the urgent necessity of establishing a system that is responsive to demand and ensures consistent expertise and quality. There are several issues that need to be considered, such as securing the number of genetic counselors to meet the needs of the field. In particular, to establish a national qualification for genetic counselors, it is necessary to implement a qualification law; to this end, further encouragement by the relevant stakeholders, including genetic counselors, is required. Discussions need to continue with various stakeholders, including government officials and lawmakers.

## Increasing the Capacity for Research as the Next Challenge for Genetic Counselors

Another challenge for genetic counselors is increasing their capacity to carry out research, as many genetic counselors work in clinical settings. In the United States, there has been an increase in the proportion of genetic counselors working in non-clinical settings such as testing companies, training programs, and research institutions (Cohen and Tucker, 2018). In Japan, there are a few genetic counselors working in non-clinical settings, and many genetic counselors are involved in various concurrent jobs. While many genetic counselors are working in teams on research projects such as clinical studies, there are only a few genetic counselors whose primary job is research.

However, to develop activities as part of the genetic counseling profession, it is necessary to evaluate and improve practice. Simultaneously, institutional and social issues—such as the position of the profession in collaboration with multiple professions and the regulation of qualifications—need to be considered. To implement these things, we believe that research is a powerful tool that can capture and evaluate phenomena from an objective standpoint and consider issues and identify solutions.

The importance of genetic counselors conducting research has already been highlighted (Biesecker, 2018). National Society of Genetic Counselors (2018)—the main professional association of genetic counselors in North America—has also listed research as one of the areas of focus for its Strategic Plan 2019–2021. Moreover, there have been world conferences since 2017 to discuss empirical research on genetic counseling, which have been attended by genetic counselors from across the globe (Middleton et al., 2019).

The Japanese Association of Certified Genetic Counselors clearly states in its Code of Ethics that research is one of its duties “to contribute to the development of professional knowledge and skills as a CGC through efforts in practice, research and education” (Japanese Board of Genetic Counseling, 2010). Although only little has been discussed on the significance of the involvement of genetic counselors in research in Japan, many genetic counselors are already conducting research in various fields such as genetic counseling and genomic medicine, and are actively making presentations at academic conferences and publishing articles. The research areas and subjects are gradually expanding, including research involving clients who have received genetic counseling (Yotsumoto et al., 2016; Watanabe et al., 2017) and studies on healthcare professionals, including genetic counselors (Yoshida et al., 2020).

However, few studies in Japan have examined the institutional and social issues surrounding genetic counselors, such as workforce issues, evaluation of genetic counseling by the public, and its cultural impact. There is limited information, such as published statistical data, on these issues. Many of them are also written in Japanese. There is limited information from other countries, especially those with a short history of genetic counselors. We believe that expanding these areas of studies will be useful in promoting an understanding of the genetic counseling profession among stakeholders such as healthcare policymakers. Future developments in this area are desirable, including collaboration with researchers in other countries, as a certain number of samples and experience will be necessary to conduct research in these areas.

As more and more healthcare professionals begin to get involved in genomic medicine, genetic counselors with specialized training programs in genetic counseling will become increasingly important in the future. As this occupation has a short history, in Japan and many other countries, it will be necessary to consider various aspects to make improvements in the future. We hope that genetic counselors will take various research initiatives and report their findings both domestically and internationally. This will stimulate discussions on various issues and lead to the development of the field.

## Conclusion

In Japan, genetic counseling is new, compared with many other healthcare professions. This means that there are also many institutional and social challenges surrounding the profession and the necessity for it to respond to a rapidly growing need among patients. During the past 15 years, the foundation of the profession has been laid based on precedents from both Japan and abroad, and it appears that genetic counselors are moving to the next step in their development as a profession, which will support the future of medical care and deliver high-quality genetic counseling to those who need it. Although there are differences in the medical systems and cultural backgrounds of all countries, we can learn a lot from the variety of efforts in other countries, such as how to tackle workforce issues and improve the quality of genetic counseling. Therefore, it will be important for Japanese genetic counselors to take the initiative in promoting discussion and creating a professional role in genomic medicine, while drawing inspiration from their global colleagues.

Ormond et al. (2018) stated that, as the role of genetic counselors is becoming more diverse in the era of precision medicine, they can learn from each other and share experiences, which they can apply to their own national context. Through research and other activities, sharing institutional and social challenges faced by genetic counselors in various countries and communicating with them will be beneficial for the development of the profession.

## Data Availability Statement

The original contributions presented in the study are included in the article/supplementary material, further inquiries can be directed to the corresponding author/s.

## Author Contributions

YA and KK discussed institutional and social issues surrounding genetic counselors in Japan and came up with the idea for the work. YA investigated the relevant literature and wrote the first draft. All authors discussed and refined the manuscript and completed it.

## Conflict of Interest

The authors declare that the research was conducted in the absence of any commercial or financial relationships that could be construed as a potential conflict of interest.
